# Buffering Caregiving Stress Through Activities: A Daily Diary Study of Dementia Caregivers

**DOI:** 10.1093/geroni/igaf122.2645

**Published:** 2025-12-31

**Authors:** Suyoung Nah, Jyoti Savla, Karen Roberto

**Affiliations:** Kyungpook National University, Blacksburg, Virginia, United States; Dept. of Human Development and Family Science, Blacksburg, Virginia, United States; Virginia Tech, Blacksburg, Virginia, United States

## Abstract

Dementia caregivers often struggle to maintain daily activities such as exercising or socializing due to caregiving demands. According to the Activation Restriction model, limitations on typical or pleasurable activities can negatively impact emotional well-being. However, it remains unclear which types of daily activities are linked to caregivers’ emotional health. We examined whether participation in religious, physical, and social activities is associated with better daily mood, and if these activities buffer the negative effects of caregiving stress on mood. We analyzed 7-day diary data from 158 dementia family caregivers (Mage = 65 ± 11.8 years; 89% White). Each day, caregivers reported their engagement in religious activities (e.g., attending church), physical activities (e.g., exercise), and social activities (e.g., talking with friends/family), as well as perceived stress from the care recipient’s symptoms. Daily mood was measured in terms of positive and negative emotions. Using multilevel models that simultaneously accounted for the three activity types, we found that only physical activity was associated with higher positive mood (β = 0.11, SE = 0.03, p = .001) and lower negative mood (β = -0.03, SE = 0.01, p = .008). Moreover, on high-stress days, physical activity mitigated the negative impact of caregiving stress on mood. That is, on days when stress levels were elevated, caregivers who engaged in physical activity experienced lower negative mood than those who did not. Findings highlight the importance of physical activity for caregivers’ emotional well-being. Interventions should incorporate strategies to include physical activity into caregivers’ daily routines.

